# Artifact Augmentation for Enhanced Tissue Detection in Microscope Scanner Systems

**DOI:** 10.3390/s23229243

**Published:** 2023-11-17

**Authors:** Dániel Küttel, László Kovács, Ákos Szölgyén, Róbert Paulik, Viktor Jónás, Miklós Kozlovszky, Béla Molnár

**Affiliations:** 1 Image Analysis Department, 3DHISTECH Ltd., 1141 Budapest, Hungary; 2 John von Neumann Faculty of Informatics, Óbuda University, 1034 Budapest, Hungary; 3 Medical Device Research Group, LPDS, Institute for Computer Science and Control, Hungarian Academy of Sciences (SZTAKI), 1111 Budapest, Hungary; 4 2nd Department of Internal Medicine, Semmelweis University, 1088 Budapest, Hungary

**Keywords:** tissue detection, convolutional neural network, deep learning, augmentation, U-Net, segmentation, classification, digital microscope scanner, digital pathology

## Abstract

As the field of routine pathology transitions into the digital realm, there is a surging demand for the full automation of microscope scanners, aiming to expedite the process of digitizing tissue samples, and consequently, enhancing the efficiency of case diagnoses. The key to achieving seamless automatic imaging lies in the precise detection and segmentation of tissue sample regions on the glass slides. State-of-the-art approaches for this task lean heavily on deep learning techniques, particularly U-Net convolutional neural networks. However, since samples can be highly diverse and prepared in various ways, it is almost impossible to be fully prepared for and cover every scenario with training data. We propose a data augmentation step that allows artificially modifying the training data by extending some artifact features of the available data to the rest of the dataset. This procedure can be used to generate images that can be considered synthetic. These artifacts could include felt pen markings, speckles of dirt, residual bubbles in covering glue, or stains. The proposed approach achieved a 1–6% improvement for these samples according to the F_1_ Score metric.

## 1. Introduction

The prevalence of digital imaging technologies is rapidly growing in routine histopathology nowadays [1,2]. The digital revolution first began in the research domain [3] with the gradual spread of whole slide image (WSI) technology due to the ease of sharing, remote visual inspection, seamless integration, image processing and analysis capabilities, efficient storage, and reliable archiving of digital slides. Various scanning devices have been developed to capture detailed tissue structures, ensuring high color fidelity and resolution through the optimal configuration of scanning parameters [4,5]. However, the need for automating and expediting the scanning processes arose as these solutions entered daily routine diagnostics where the substantial volume of samples hinders individualized fine-tuning of scanning parameters per slide, necessitating intelligent tissue detection algorithms [6].

**Figure 1:**
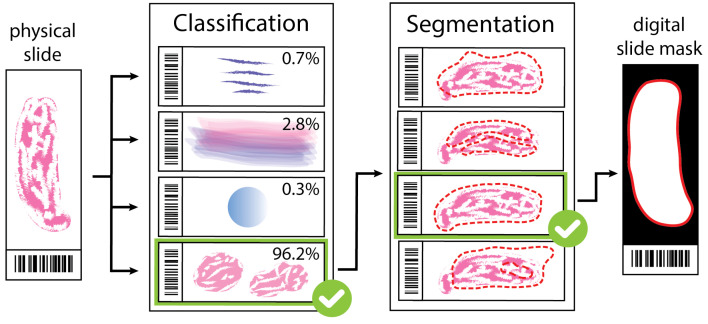
An illustration of a 2-step tissue detection protocol. First, a classification algorithm identifies the sample by selecting the most probable class from a predefined set on which the model was trained. Then, the class-type specific segmentation algorithm detects the exact location of the specimen on the slide by outlining it with a contour mask. In this protocol, each class has a corresponding segmentation algorithm which is the most optimal in detecting the morphological features of the given class.

To tackle this challenge, scanning is usually preceded by taking a preview image (only a few megapixels) of the entire slide during the loading process in most WSI systems [4,7]. Afterward, tissue detection algorithms are employed to recognize both the sample type and its position (see Figure 1). The accurate localization of the specimen influences crucial aspects of WSI capture, including scanning time, focus quality, the effectiveness of compensation algorithms, and ultimately, accurate diagnosis. To meet these criteria, deep learning-based tissue detection solutions [8] have recently emerged as increasingly successful approaches in sample segmentation and localization [9].

State-of-the-art convolutional neural networks (CNNs) require a large amount of training and validation data to ensure reliable performance in tissue detection [10]. The paucity of diverse and representative sets of training images may act as a bottleneck in improving the algorithm’s prediction accuracy. To circumvent this limitation, strategies such as data augmentation emerge as pivotal aids [11,12]. Most common image augmentation techniques include geometric transformations (scaling, rotating, mirroring, cropping, zooming) [13], stain color augmentation [14,15], random erasing [16], noise injection [17], image style transfer [18], and copy–paste augmentation [19,20].

Several solutions have been developed to implement these augmentation concepts. Albumentations, for example, is a flexible and fast open-source approach that offers a rich variety of image transforms for classification, segmentation, and detection [21]. Stainlib provides methods for color augmentation and normalization of histopathology images [22] and also includes deep learning-based approaches that perform stain-invariant training of CNN models. RandAugment is an automated data augmentation method that involves various image transformations with the reduced need for search space [23]. Domain-specific modifications to the RandAugment framework could provide even better results [24] than the state-of-the-art manually engineered data augmentation strategies on H&E-stained histopathology slides from the Camelyon17 dataset [25]. Conditional generative adversarial networks, like HistoGAN [26], are also competent in synthesizing realistic histopathology image patches for augmentation purposes conditioned on class labels to mitigate the challenges of supervised training for automated histopathological classification. Another example is an object-blend method called Soft-CP for semantic segmentation of medical lesions that combines the copy-paste augmentation method to solve the problem of scarcity and data imbalance in medical datasets [27].

**Figure 2:**
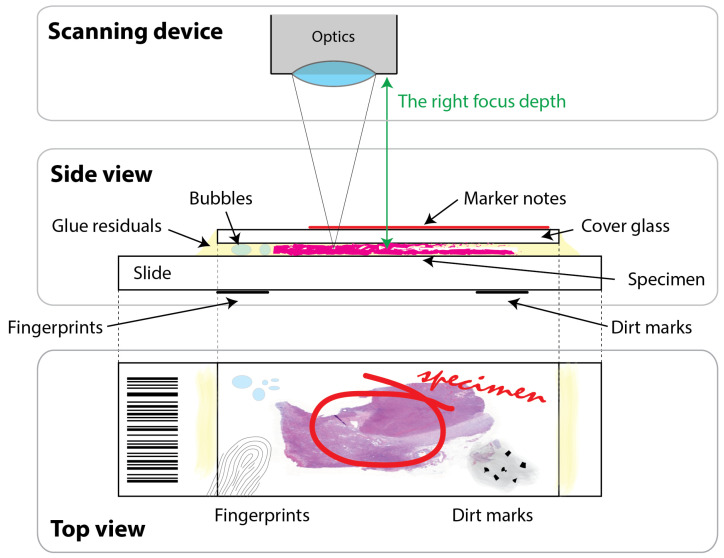
The figure illustrates that many artifacts may ruin finding the right focus depth in whole slide imaging. The top two blocks show the relative position of the scanning device’s optics and the histology slide with its different layers, i.e., glass slide; specimen; cover glass; covering glue; possible air bubbles in the glue; and dirt, fingerprint, and marker artifacts on the glass surfaces. The bottom block shows the top view of the histology slide. The illustration is not to scale.

From the WSI scanning point of view, one critical aspect of tissue detection is the precise discrimination of specimens from imaging artifacts in slides: some samples may be yellowed, dirty, have fingerprints or felt pen markings on them, or contain bubbles in the covering glue. The precise sample localization is not only important for selecting relevant areas for diagnosis but also essential for improving focus and image quality: focusing must only be performed in the sample areas (Figure 2). The focus depth difference between layers of a typical histology slide can be as big as 100 µm or more. Thus, an accidental misfocusing on a felt pen marking on the top of a cover glass, for example, may lead to a completely obscure scan image of the sample area. Similarly, knowing the exact location of the sample is also essential to calculate the compensation images. Thus, augmenting the training and validation dataset with these specific types of imaging artifacts can significantly improve the preview detection algorithm’s performance in WSI scanning systems.

**Figure 3:**
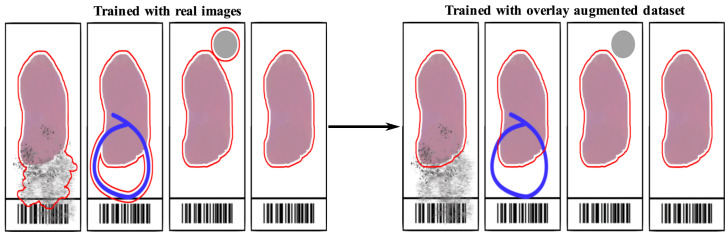
The overlay augmentation of imaging artifacts (specks of dirt, felt pen marks, bubbles) may significantly increase the accuracy of tissue detection in histology slides. The drawing illustrates the results of a tissue detection algorithm with and without overlay augmentation of various artifacts on the left-, and on the right-hand side, respectively.

In this paper, we give a solution to the problem of tissue detection in histology slides with non-negligible imaging artifacts by introducing a new overlay augmentation method. The augmentation is performed by randomly placing various types of artifact layers on top of originally clean preview images. These artifacts may include speckles of dirt, air bubbles stuck in the covering glue, felt-tip markings on the glass, and brand markings (Figure 3). This type of augmentation enables the synthetic diversification of the training and validation set of preview images to prepare the tissue detection models for better scanning performance during routine diagnostics in cases when these artifacts cannot be avoided. Our solution thus allows for a controlled augmentation of the dataset by generating samples that can be considered as synthetic, while also optimizing the resources needed to perform the task by eliminating the need to label new samples.

## 2. Materials and Methods

Most WSI microscope scanners contain a low-resolution preview camera— of a few megapixels—for preliminary identification and recognition of the sample. This resolution is sufficient to scan the bar or QR code at the bottom of the slide, as well as to detect the type and location of the tissue sample. When the slide is loaded in, the preview image is captured first, then the scanner’s tissue detection algorithm determines which areas need to be scanned. Then the scanner system defines a strategy for the scanning path and selects the focusing points and the location of the compensation images. All of these must be faultless for perfect high-resolution digitization. In addition, the sample should be detected as narrowly as possible to increase efficiency; however, leaving out even a small fraction of the sample area is not acceptable to prevent potential misdiagnosis.

Thus, in this section, we will start by providing a detailed description of the overlay augmentation algorithm. Following that, we will present the U-Net architecture used for training the model along with a brief overview of the training circumstances, and then discuss the details of constructing the training dataset.

### 2.1. Overlay Augmentation

**Figure 4:**
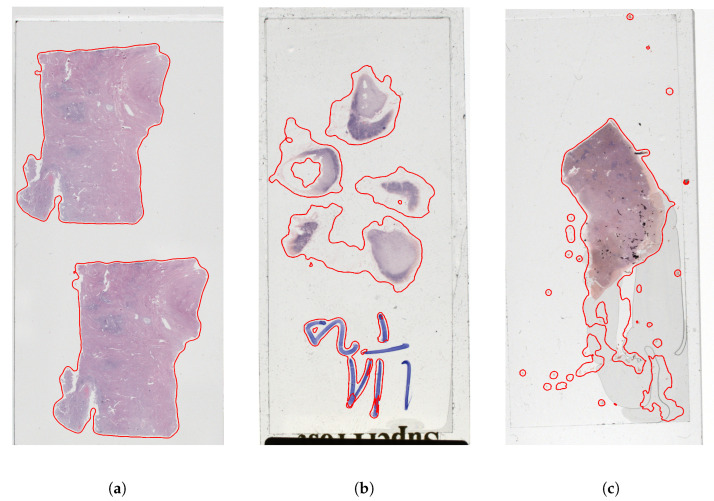
Accurate segmentation and missegmentation of the objects. (**a**) Accurate segmentation. (**b**) Felt pen error. (**c**) Bubble error.

During the training of a U-Net model, it is essential to avoid both underfitting and overfitting. Thus, the optimal cardinality of representative images is crucial in the training set. We experienced that the paucity of available preview images (especially those with imaging artifacts) significantly decreased the prediction accuracy of models that are later used on images with speckles of dirt, air bubbles trapped under the cover glass, felt pen markings on the glass, and brand markings; see, e.g., Figure 4.

To overcome this challenge and to improve the performance of the model, we propose to add synthetic artifacts to originally clean images by which we augment the training set of images. We developed a copy–paste method [19,20] in which images of artifacts like felt pen, bubbles, or dirt (or a combination of these) are copied onto the original preview images of tissue samples. Our approach to overlay augmentation can be divided into three major steps: First, we prepare instances of imaging artifacts that we collected from dirty or faulty glass slides, which is detailed in Section 2.1.1. Then, we change the appearance of the artifact with different transformations and generate multiple variations of them (Section 2.1.2). Finally, we randomly place one or more synthetic overlays of artifacts on clean slides (Section 2.1.3).

#### 2.1.1. Labeling of Artifacts in the Images

**Figure 5:**
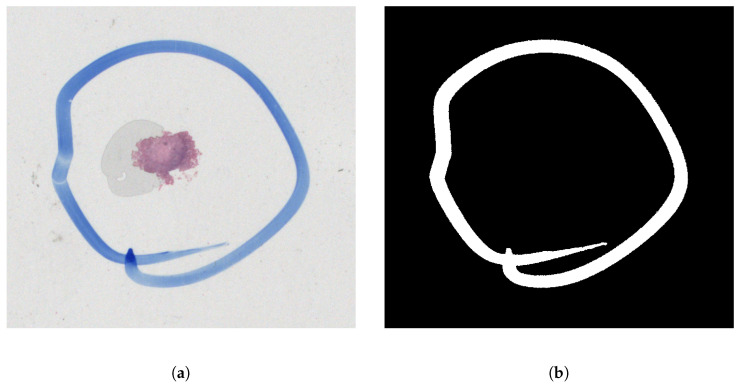
Labeling of the overlay. (**a**) Original image. (**b**) Mask of felt pen.

The augmentation process begins with the collection and manual labeling of artifacts in slide images using an image editing software, Krita (https://krita.org/, accessed 9 January 2023). The labeling is similar to the annotation of preview images using multilayer TIF files mentioned above. The new mask image has the same size as the original image and the region of interest (ROI) is marked in white while the background is in black (see Figure 5).

#### 2.1.2. Augmentation of Overlay Images

**Figure 6:**
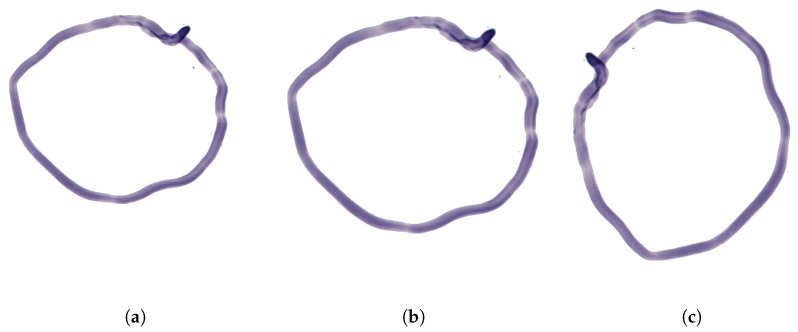

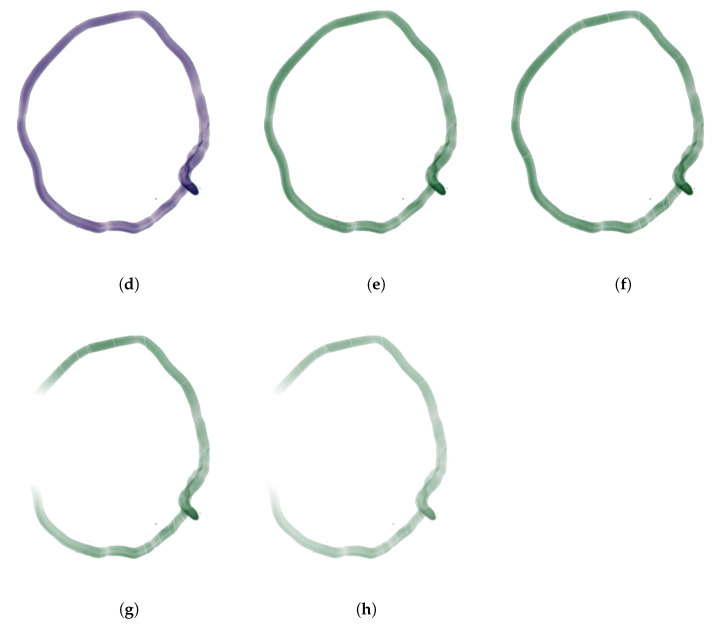
Steps of overlay augmentation. (**a**) Without augmentation. (**b**) Scaling. (**c**) Rotation. (**d**) Mirroring. (**e**) Changing of hue. (**f**) Scratch. (**g**) Perlin noise. (**h**) Intensity scaling.

The images of artifacts are augmented to increase the diversity of their appearance before combining them as “overlay images” with the clean preview images of tissue samples. The augmentation may comprise both geometric transformations like rescaling, rotating, and mirroring, and content transformations like changing hue, alpha blending, or adding synthetic scratches and noise (see Figure 6). Each transformation can be parameterized with one or more random factors to ensure that the output overlay is slightly different from the others. Whether the overlay transformation is used or omitted is optional and depends on the type of the original histology slide and the user’s purposes. The applied transformations are performed with the OpenCV library [28] and include the following:

**Scaling**—a uniform linear dilation with a random scaling factor of a value between 0.75 and 1.25.
**Rotation**—rotates the image with a random integer angle in the range of $\left[0^{\circ },359^{\circ }\right]$. No cropping is applied after the rotation, meaning the size of the image is not preserved. The non-image areas are filled with zero pixel values.
**Mirroring**—flips the image along a specified axis or axes. There are three variations of mirroring: horizontal, vertical, or both.

Felt pen overlay transformations include:

**Hue**—changes the hue of all pixels of a felt pen marking to a random integer picked from [0,180] in HSV color space representation. The RGB to HSV conversion enables independent manipulation of the hue component while preserving the saturation and value components.
**Scratch**—adds a scratching effect to felt pen overlays. It draws a random number of one-pixel-thick straight lines between the edges of the image at random angles. The lines represent a random multiplication factor between 0 and 0.2 with which the pixel intensities are reduced where the lines intersect the felt pen markings on the slide. For a more realistic appearance, the overlay image of the lines is blurred with a 3×3 kernel before applying it on the felt pen overlay.
**Perlin noise**—using the PerlinNoise package (https://github.com/salaxieb/perlin_noise, accessed 8 June 2023), it generates a Perlin noise-weighted subtraction layer to felt pen overlay, optionally applying thresholds and normalization which may smooth the edges of the layer. Practically, this transformation fades random regions in felt pen overlay images.
**Intensity scaling**—rescales the intensity of the felt pen overlay image by a random number sampled from a uniform distribution in a range between 0.3 and 0.7 and raised to the power of 0.5. (Note that the negative of this overlay is subtracted from the original preview image.)

These transformations can be applied independently during the overlay augmentation process.

#### 2.1.3. Combining Synthetic Overlays with Preview Images

**Figure 7:**
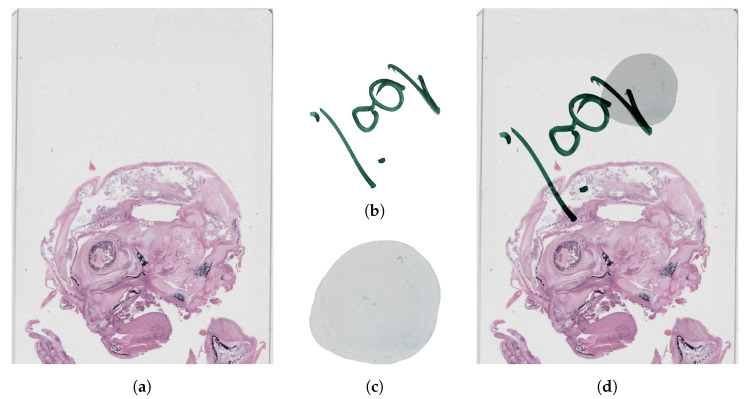
Artifact addition to an originally clean preview image. (**a**) Part of the input image. (**b**) Felt pen overlay. (**c**) Bubble overlay. (**d**) Part of the output image.

The last step in overlay augmentation is to combine the synthetic overlays with the original preview images of WSIs. In the case of bright field microscopy, the correct way of combining images is to subtract the negative of the artifact overlay from the preview image to preserve the original visual appearance for both, as in Figure 7. It is important to subtract the artifact only and not the entire image so the negative of the artifact overlay is masked, having zero pixel intensities for the background.

The masked overlay image ($I_{\mathrm{over}}$) is subtracted from the “input” preview image ($I_{\mathrm{in}}$) as

(1) $$I_{\mathrm{out}}\left(x,y,c\right)=\max\left(0,I_{\mathrm{in}}\left(x,y,c\right)+I_{\mathrm{over}}\left(\tilde{x},\tilde{y},c\right)-255\right))$$

where images are represented in N×M×3 arrays with pixel indices *x* and *y* running from 0 to either *N* or *M*, where *N* is the width and *M* is the height of the input image, while $\tilde{x}$ and $\tilde{y}$ are pixel indexes of the overlay. *c* indicates the 3 RGB color channels. If the overlay image is narrower than the preview image, i.e., $\max(\tilde{x})\leq N$ then $\tilde{x}\in \left[x_{0},x_{0}+\max\left(\tilde{x}\right)\right]$ for any $x_{0}$ such that $x_{0}\leq N-\max\left(\tilde{x}\right)$. Similarly, if it is shorter than the preview image, i.e., $\max(\tilde{y})\leq M$ then $\tilde{y}\in \left[y_{0},y_{0}+\max\left(\tilde{y}\right)\right]$ for an arbitrary $y_{0}$ such that $y_{0}\leq M-\max\left(\tilde{y}\right)$. In the opposite case when the overlay image is wider than the preview image, it is cropped horizontally as $\tilde{x}\in \left[x_{0},x_{0}+N\right]$ for an arbitrary $x_{0}$ such that $x_{0}\leq \max\left(\tilde{x}\right)-N$; similarly, if it is taller, it is cropped vertically as $\tilde{y}\in \left[y_{0},y_{0}+M\right]$ for a random $y_{0}$ such that $y_{0}\leq \max\left(\tilde{y}\right)-M$.

The advantage of this overlay augmentation method is that the higher-intensity pixels are always kept from either the original preview image or the overlay image. In addition, the corresponding area outside of the artifact in the overlay remains unchanged in the output image.

### 2.2. Training Process with Overlay Augmentation

**Figure 8:**
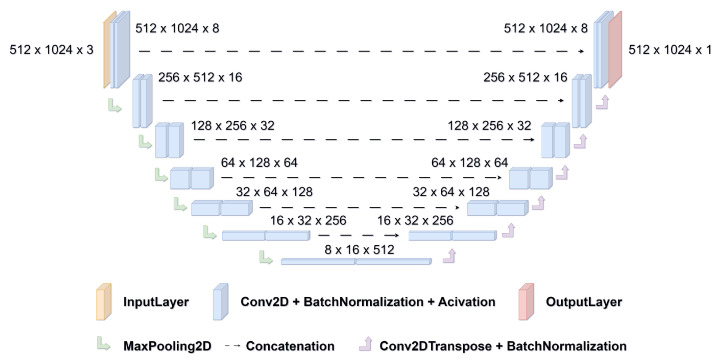
The U-Net architecture consists of a contracting path of 7 layers that captures contextual information and a symmetric expansive path of another 7 layers that recovers spatial details. The layers are interconnected with 2D max-pooling in the contracting branch, and we applied 2D convolutional transpose with batch normalization between the layers on the expanding branch.

Our tissue detection solution utilizes a convolutional neural network model with a standard U-Net architecture [29], which is configured as follows (Figure 8): it comprises an input layer, succeeded by 6 encoder blocks. Each encoder block consists of two convolution blocks and a max-pooling layer, with batch normalization and ReLU activation for feature extraction. On the decoder side, we mirror the encoding process with convolution blocks and Conv2DTranspose layers for dimension expansion. Skip connections between encoder and decoder enhance feature preservation and localization. This enables the model to harness low-level and high-level information effectively during the segmentation process, contributing to its remarkable performance in tissue detection tasks.

The tissue detection model is trained with annotated preview images in a reinforced learning fashion. The images are annotated by a histology professional using multilayer TIF files. In the file, the first layer is the native preview image and the second layer is a binary mask where white and black pixels represent the specimen and the background, respectively. The model learns to segment images according to the given masks. After several training epochs, the model successfully converges and is capable of predicting sample outlines.

The training can be divided into two steps: a preparation and a training phase. To commence the preparation step, we reserved a fixed portion of the training data for testing purposes ensuring that the model’s performance could be rigorously evaluated on unseen samples later.

The remaining training data underwent a series of preprocessing steps to prepare it for training. Initially, each image was resized to a fixed dimension of 512×1024 pixels, subsequently, various data augmentation techniques were applied to augment the training dataset. Despite the use of traditional augmentation techniques during the training process, there remains a scarcity of images featuring artifacts in the training set. This shortage necessitates the utilization of the overlay augmentation method to further improve the prediction accuracy for these types of slides. It should be mentioned that the augmentation method we proposed is not limited to the U-Net architecture employed in our implementation; it can be effectively applied to various other neural network architectures for image analysis tasks.

During training, the preprocessed data was split into an 80:20 ratio, with 80% allocated for training and 20% for validation. This division allowed us to monitor the model’s performance and assess its generalization using the Intersection over Union (IOU) metric on the validation set, ensuring comprehensive progress tracking. This metric served as a valuable indicator for determining when to halt the training process, ensuring that the model achieved the desired level of accuracy and robustness. Additionally, to strike a balance between computational efficiency and gradient accuracy, we employed a batch size of 16 during the training process. The duration of the training process depends to a large extent on the number of epochs and the amount of augmentation. In our case, it took an average of 10–15 epochs per learning cycle to achieve the best results.

### 2.3. Training Data

Routine anonymized slides were used as training data from the archive of the 1st Department of Pathology and Experimental Cancer Research of Semmelweis University, Budapest, Hungary. Different stained samples were randomly collected, and the specimens were deidentified and did not contain any details of the patient. The samples were used for a retrospective study without impacting patient care.

Slides were digitized using a Pannoramic^®^ P250 Flash and Pannoramic^®^ P1000 digital slide scanners (3DHISTECH Ltd., Budapest, Hungary) that utilizes Plan-Apochromat objective with 20× or 40× magnification (Zeiss, Jena, Germany) and a CIS color camera (CIS Corporation, Tokyo, Japan) for brightfield image acquisition.

During the training, only images digitized by the scanners in a preview manner were used, the average resolution of which was around 4 Megapixels. The full-resolution whole-slide image was only used to help and validate the labeling process. The resulting dataset consisted of 937 image pairs.

## 3. Validation

The validation of the proposed method is based on an independent ground-truth dataset marked by experts, on which the same labeling method is used as during teaching. The prediction is performed on the input image and compares the output mask to the ground-truth mask. The more the two match, the higher the accuracy of the method.

Since the proposed method improves the most in the case of samples containing artifacts, the following samples were selected from the archival pathology sample set by random sampling, which was mostly stained with Hematoxylin and Eosin (H&E), Progesterone, HER2, and Ki67 reagents:

- Samples without artifacts: 116;
- Samples with felt-pen markers: 58;
- Samples with bubbles: 86;
- Samples with dust: 56.

During the validation, the performance of the U-Net model without synthetic samples is compared with the proposed method that also uses overlay-augmented samples. In order to make the comparison as objective as possible, several types of metrics are used; according to our experience, using the Sensitivity (Equation (2)), Precision (Equation (3)), and F_1_ Score (Equation (4)) metrics, the goodness, false negatives, and false positives can also be measured well. Each of the metrics is based on the number of true positive (*TP*), false positive (*FP*), true negative (*TN*), and false negative (*FN*) pixels between the two masks.

(2) $$Sensitivity=\frac{TP}{TP+FN}$$

(3) $$Precision=\frac{TP}{TP+FP}$$

(4) $$F_{1}Score=\frac{2TP}{2TP+FP+FN}$$

## 4. Results

Table 1 shows the average results of the training containing only original images and the results using synthetic samples, with Sensitivity, Precision, and F_1_ Score metrics.

Examining the numerical results, it can be seen that the segmentation results improved in almost all cases using synthetic images. In the case of artifact-free samples, the improvement was only minimal. In terms of the F_1_ Score metric, the improvement was around 1% (0.9091 ⇒ 0.9175), while in the case of the felt pen and bubbled samples, the improvement was the largest: 4.2% in the case of felt pen samples (0.8491 ⇒ 0.8853) and 5.9% in the case of bubbled samples (0.7528 ⇒ 0.7970). In the case of dirty samples, only a small improvement was observed, around 1% (0.8839 ⇒ 0.8921).

Table 2 shows the standard deviations of results calculated from Sensitivity, Precision, and F_1_ Score metrics.

From the standard deviation values, it can be deduced that the variability of the quality of the results decreases in almost all cases when the proposed augmentation step is applied. Regarding the F_1_ Score, the standard deviation is between 0.13 and 0.20 without the proposed augmentation step, while when using overlay augmentation it decreases to between 0.08 and 0.17. In the case of samples with bubbles, the degree of improvement is particularly high (0.1592 ⇒ 0.0764).

**Figure 9:**
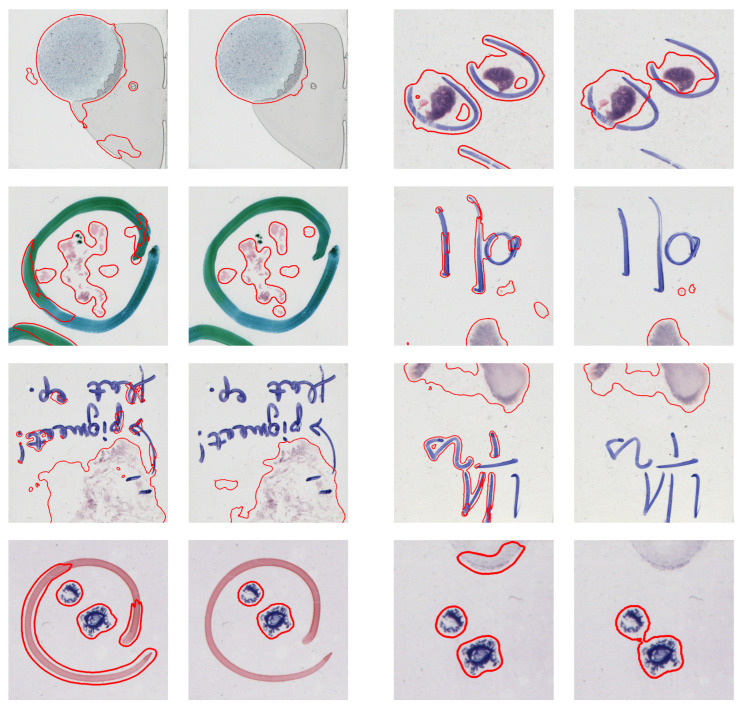
Illustration of the detection results, trained with the original training set (first and third column) and extended with the synthetic samples (second and fourth column).

Figure 9 shows the segmentation results before and after overlay augmentation. The segmentation results are significantly better in the areas where there is an artifact pattern that was included in the training set during overlay augmentation.

**Figure 10:**
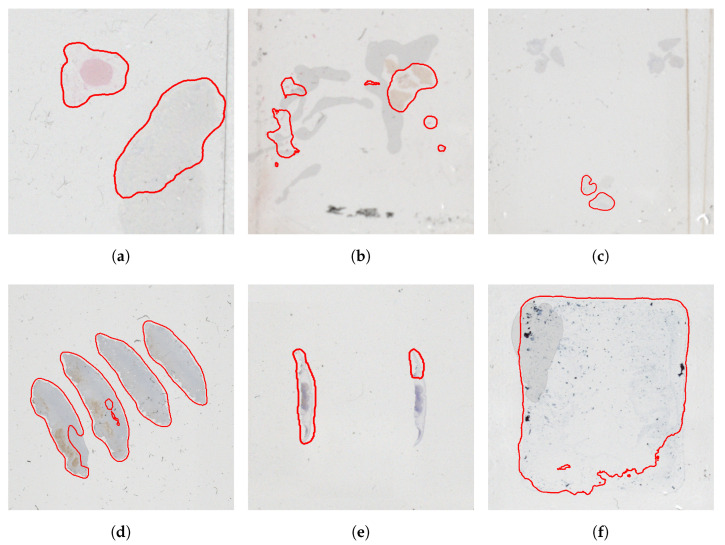
Illustration of cases where detection is still not performed correctly using overlay augmentation: (**a**) part of the bubble was identified as tissue, (**b**–**d**) missed sample—detected as bubble, (**e**) missed sample—detected as felt-tip pen, (**f**) missed sample—detected as dirt.

There are cases when overlay augmentation cannot help the result of the segmentation either. Figure 10 shows some such cases. In sub-figure (a), compared to the area of the sample (upper-left part), the size of the bubble is too large and it looks similar to the sample; in this case the augmentation could only partially help. In sub-figures (b–d) the tissue sample was omitted; as the tissues look very similar to the bubble, the algorithm incorrectly cut out parts of the tissue sample. Sub-figure (e) shows a needle biopsy sample where the tissue sample resembles a felt-tip marker and the algorithm incorrectly detected it as a felt-tip marker. The right-bottom part of sub-figure (f) was mistakenly identified as dirt and cut off from the sample area. In the cases of these samples, an improvement could probably be achieved by further increasing the number of training and overlay augmentation samples.

## 5. Discussion

Our investigation delved into the integration of synthetic samples into the training dataset to bolster the performance of a tissue sample segmentation model. The present results suggest that by augmenting the images with felt-tip pens, bubbles, and dirt, in terms of F_1_ Score, the proposed method achieved a 1% enhancement in the case of artifact-free and dirty samples, while in the cases of images with felt-tip pens and bubbles the improvement was between 4% and 6%. Based on the standard deviation values, not only did the goodness increase, but the detection could also be more confident with the help of the proposed augmentation step.

However, it is important to mention that the effectiveness of the proposed method is highly sample-dependent. The method shows no significant improvement on ideal samples that do not contain anything other than the tissue sample in the scanned image. On the other hand, in cases where the preparation of the sample is not adequate, the sample is archival or something has been marked on it with a pen, the method can improve it to a large extent.

While analyzing the results of the validation set, several ideas emerged for the further development of the proposed methods. One such improvement could be adjusting the amount of overlay augmentation to the tissue type. There are samples that very rarely contain, for example, felt-tip markings, such as tissue microarray samples and full-slide smears, in which case, felt-tip augmentation should not be used on these samples. For this, in the training set, each sample should be assigned which overlay augmentation steps can and should be applied to them.

Another improvement option is the use of generative adversarial networks (GANs), including conditional GANs (cGANs), which are particularly useful for tasks such as image-to-image translation, where the goal is to generate images based on certain attributes or features. This enhancement is expected to increase the variability of the training data further since the generated images or image details can now be fully classified as synthetic.

In addition to digital microscopy, the proposed method can be adapted to other disciplines, where the training set requires images that were produced under several circumstances and thus contain artifacts. The field of application can be remarkably diverse, and the proposed method is flexible enough to be transferred with few modifications.

## 6. Conclusions

The objective of this study was to explore the augmentation of a learning-based tissue sample segmentation method, especially in scenarios where access to ample training data is constrained due to the arduous nature of data acquisition and where conventional approaches struggle to establish model robustness.

This study has introduced a novel approach to enhance the performance of learning-based tissue sample segmentation methods by augmenting the training dataset with synthetic samples. Our experiments have demonstrated that by integrating artifacts such as felt-tip pen markings, bubbles, and dirt into the training data, significant improvements in segmentation accuracy can be achieved, particularly in scenarios where samples contain artifacts.

Finally, we can conclude that, based on the validation dataset, the method can mostly eliminate detection artifacts that may resemble the sample, such as felt-tip pens and bubbles. The method can be extended to other samples that contain other kinds of artifacts, and it can also be adopted to other disciplines. 

## Figures and Tables

**Table 1:** Average results without and with using synthetic images in training U-Net models.

Algorithm	Sample Category	Sensitivity	Precision	F_1_ Score
Traditional augmentation	Artifact-free	0.8728	0.9486	0.9091
	Felt pen	0.7831	0.9272	0.8491
	Bubbled	0.6138	0.9732	0.7528
	Dirty	0.8473	0.9237	0.8839
Proposed augmentation	Artifact-free	0.8895	0.9473	0.9175
	Felt pen	0.8337	0.9437	0.8853
	Bubbled	0.6749	0.9729	0.7970
	Dirty	0.8587	0.9283	0.8921

**Table 2:** Standard deviations of metrics without and with using synthetic images in training U-Net models.

Algorithm	Sample Category	Std. Sens.	Std. Prec.	Std. F_1_ Score
Traditional augmentation	Artifact-free	0.8728	0.9486	0.9091
	Felt pen	0.0985	0.1473	0.1362
	Bubbled	0.1373	0.1956	0.1592
	Dirty	0.1946	0.1547	0.1956
Proposed augmentation	Artifact-free	0.1275	0.1151	0.1188
	Felt pen	0.0729	0.1018	0.0827
	Bubbled	0.1011	0.0879	0.0764
	Dirty	0.1773	0.1616	0.1727

## Data Availability

Data used in this study can be made available upon reasonable request.
